# Comparative Proteomic Analysis of Carbonylated Proteins from the Striatum and Cortex of Pesticide-Treated Mice

**DOI:** 10.1155/2015/812532

**Published:** 2015-08-09

**Authors:** Christina Coughlan, Douglas I. Walker, Kelly M. Lohr, Jason R. Richardson, Laura M. Saba, W. Michael Caudle, Kristofer S. Fritz, James R. Roede

**Affiliations:** ^1^Department of Pharmaceutical Sciences, Skaggs School of Pharmacy and Pharmaceutical Sciences, University of Colorado, Aurora, CO 80045, USA; ^2^Department of Medicine, Emory University, Atlanta, GA 30322, USA; ^3^Department of Environmental Health, Rollins School of Public Health, Emory University, Atlanta, GA 30322, USA; ^4^Department of Environmental and Occupational Medicine, Rutgers University Robert Wood Johnson Medical School, Piscataway, NJ 08854, USA

## Abstract

Epidemiological studies indicate exposures to the herbicide paraquat (PQ) and fungicide maneb (MB) are associated with increased risk of Parkinson's disease (PD). Oxidative stress appears to be a premier mechanism that underlies damage to the nigrostriatal dopamine system in PD and pesticide exposure. Enhanced oxidative stress leads to lipid peroxidation and production of reactive aldehydes; therefore, we conducted proteomic analyses to identify carbonylated proteins in the striatum and cortex of pesticide-treated mice in order to elucidate possible mechanisms of toxicity. Male C57BL/6J mice were treated biweekly for 6 weeks with saline, PQ (10 mg/kg), MB (30 mg/kg), or the combination of PQ and MB (PQMB). Treatments resulted in significant behavioral alterations in all treated mice and depleted striatal dopamine in PQMB mice. Distinct differences in 4-hydroxynonenal-modified proteins were observed in the striatum and cortex. Proteomic analyses identified carbonylated proteins and peptides from the cortex and striatum, and pathway analyses revealed significant enrichment in a variety of KEGG pathways. Further analysis showed enrichment in proteins of the actin cytoskeleton in treated samples, but not in saline controls. These data indicate that treatment-related effects on cytoskeletal proteins could alter proper synaptic function, thereby resulting in impaired neuronal function and even neurodegeneration.

## 1. Introduction

Epidemiological studies of the past two decades have implicated environmental chemicals as possible causative factors for the development of Parkinson's disease (PD) [1–4]. Due to these observations, an environmental hypothesis of PD was derived stating that there are chemicals in the environment capable of selectively targeting the dopaminergic neurons of the substantia nigra (SNpc) [5]. In a recent paper, Costello et al. determined that geographical areas with high use of the pesticides maneb (MB) and paraquat (PQ) had a high prevalence of PD [1]. In addition, PQ in combination with the dithiocarbamates, ziram and MB, gives rise to an 80% increased risk of PD [6]. During the past decade, a number of studies have been conducted investigating the effects of PQ and MB on dopaminergic neurons* in vivo*. For example, the majority of these studies have shown that the combination of PQ and MB causes decreased striatal dopamine, decreased counts of tyrosine hydroxylase positive neurons in the SNpc, and impaired motor activity [7–12]. Increased lipid peroxidation has also been reported in this model [7, 13–15]. Additionally,* in vitro* studies have demonstrated that PQ and MB can alter cellular redox status [16, 17].

Oxidative stress is defined as an imbalance between oxidant production (free radicals and two-electron nonradicals, such as hydrogen peroxide) and antioxidant defenses (catalase, superoxide dismutase, peroxiredoxins, and glutathione peroxidase) [18]. There is strong evidence that points to the involvement of oxidative stress in the pathogenesis of PD. For example, etiologic factors like mitochondrial dysfunction, excitotoxicity, and inflammation all involve the production of reactive oxygen species (ROS). Additionally, postmortem analyses of PD brains have shown increased levels of iron, deficits in mitochondrial complex I activity, depleted glutathione (GSH), and increased lipid peroxidation [19–21].

Many reactive species are generated via the process of ROS-stimulated lipid peroxidation. This process can result in the accumulation of reactive aldehydes, such as 4-hydroxynonenal (4HNE). Reactive electrophiles covalently modify cellular macromolecules like proteins, DNA, and lipids. Of great importance to this study, lipid-derived electrophiles target nucleophilic amino acid residues, such as cysteine, histidine, and lysine [22–24]. This adduction leads to irreversible protein carbonylation, modulation of a protein's function (e.g., modification of the active site) [25–29], and/or aggregation and removal of the protein by cellular quality control mechanisms [28]. Additionally, free radicals can directly oxidize amino acid side chains and also lead to adduction by glycation products [30]. These events, as well as aldehyde adduction, all result in increased levels of protein carbonyls. Lastly, alterations in redox signaling and control during states of uncontrolled oxidant production and lipid peroxidation can ultimately lead to cellular toxicity and apoptosis [18].

Various investigators have published on the effects of PQ and MB in the mouse brain [9–11, 31]; however, the role of protein posttranslational oxidative modifications as an underlying factor for the initiation and progression of pesticide-induced PD remains unknown. Therefore, we performed a comparative proteomics study to investigate a brain region known to be affected by PQ and MB, the striatum, as well as a lesser-studied region, the cortex. Upon examination, after 6 weeks of biweekly injections, striatal dopamine (DA), 3,4-dihydroxyphenylacetic acid (DOPAC), and homovanillic acid (HVA) were reduced and olfactory discrimination was altered. Protein extracts from the striatum and cortex showed distinct patterns of 4HNE modification. Cortex and striatal samples, when examined further for carbonylated proteins using LC-MS/MS-based proteomics, showed distinct toxicant-related effects. While PQ affected the striatum, as evidenced by a greater number of carbonylated proteins, MB treatment yielded the greatest number of carbonylated proteins in the cortex. Proteomic pathway analyses of these striatal and cortical adducted proteins showed an enrichment of many different KEGG pathways, particularly those involving the actin cytoskeleton. Together the results demonstrate that PQ and MB exposure affect olfactory discrimination, striatal catecholamine metabolism, and increased carbonylation of proteins involved in cytoskeletal dynamics.

## 2. Materials and Methods

### 2.1. Animal Treatments

All animal procedures were performed under a protocol approved by the Institutional Animal Care and Use committee of Emory University. Male C57BL/6J mice (12 per group) were purchased from Jackson Laboratories and allowed to acclimate for one week prior to dosing. Animals were group housed in a group of 4 mice/cage and all treatment groups were present in each cage grouping. Mice were treated with saline (0.9%), PQ (10 mg/kg), MB (30 mg/kg), or the combination of PQ and MB twice per week via i.p. injection, with a total injection volume not exceeding 0.25 mL. These dose levels were chosen based upon previously published studies utilizing this model [7–11]. Animals receiving both PQ and MB received separate injections of PQ and MB. All animals were weighed biweekly prior to dosing, and prior to sacrifice. Two weeks prior to the end of the study, all animals were separated and singly housed so animals could acclimate to their own cage for olfactory discrimination testing. At the completion of all behavioral testing, animals were euthanized via live decapitation. The striatum and cortex were dissected from the brains and snap frozen in liquid nitrogen until analysis. During the course of the study, animals in all groups gained weight and no significant differences in body weight were observed (data not shown). It must be noted, however, that two animals in the PQMB group died prior to the completion of dosing (week 3 and week 5), but no deaths were observed in the groups receiving PQ or MB alone.

### 2.2. Olfactory Discrimination

Olfactory discrimination was performed with modifications as previously described [32]. In summary, individual wooden blocks were placed in 50 mL conical tubes containing 1 g of soiled bedding from either the test animals' cages or strangers' cages. Blocks were placed in the tubes for 24 h. For testing, the test mouse was presented with two blocks on opposing sides of the test cage: (1) a block scented with its own bedding and (2) a block scented with a stranger's bedding of the same sex. To determine the animal's ability to discriminate the scent of its home cage and a stranger's cage, the time spent in contact with each block was recorded for the 2 min trial. All trials were coded, videotaped, and timed by a blinded scorer. A total of 6–8 animals per treatment group were examined.

### 2.3. Locomotor Activity

All animals (10–12 per group) were tested for treatment-related impact on locomotor activity. Mice were placed in polycarbonate locomotor boxes (25.4 × 50.8 × 25.4 cm), and horizontal distance was quantified over time using Noldus Ethovision 3.0 (Noldus Information Technology, Wageningen, Netherlands) [33, 34]. General locomotion for treated and untreated mice was observed for a total of 4 h. The first 30 min was considered the habituation period to ensure stabilization of the horizontal activity signal.

### 2.4. High Performance Liquid Chromatography (HPLC) Determination of Striatal Neurochemistry

HPLC analyses of neurochemistry were performed using electrochemical detection as previously described [33, 35]. Monoamine standards for DA, dihydroxyphenylacetic acid (DOPAC), and homovanillic acid (HVA) were purchased from Sigma. Mobile phase was obtained from ESA Inc. (Chelmsford, MA). Briefly, dissected striata from test animals (6–8 per group) were sonicated in 0.1 M perchloric acid. Homogenates were centrifuged at 15,000 ×g and the supernatant was filtered through a 0.22 *μ*m filter by centrifugation at 15,000 ×g. The supernatants were analyzed for levels of DA, DOPAC, and HVA. Quantification was made by reference to calibration curves made using individual standards.

### 2.5. SDS-PAGE and Western Blot

50 *μ*g of each protein lysate (striatum and cortex) were separated via SDS-PAGE utilizing a 10% polyacrylamide gel. Transfer of the proteins onto PVDF membrane was performed using the BioRad Turbo Blotter with Trans-Blot Turbo 5x Transfer Buffer (BioRad). Blotting was performed on the mixed mw setting (8 min). Blocking was performed for 20 min in Blotto (0.5% nonfat dried milk/TBS-0.1% Tween). Primary antibodies against 4HNE (generous gift from Dr. Dennis Petersen, University of Colorado) were diluted 1 : 1000 in Blotto and incubated with the blot overnight at 4°C. After three washes for 10 min in 0.5% TBS/0.1% Tween, the blot was incubated with a horseradish peroxidase conjugated secondary antibody at 1 : 5000, diluted in Blotto. Clarity Western ECL Substrate (BioRad) was used to detect the HRP of the secondary antibody. Imaging was performed on the ChemiDoc MP imaging system and Image Lab software (BioRad). This experiment was conducted in three independent replications, and the image is a representative sample.

### 2.6. Biotin Hydrazide Derivatization of Carbonylated Protein

Hydrazide chemistry was employed to derivatize protein carbonyls from the samples (3-4 samples per treatment group) [36]. Mouse striatum and cortex lysates (400 *μ*g) were incubated in the dark with 5.0 mM biotin hydrazide (Pierce, Rockford, IL) for 2 hours at 25°C with gentle rotation. Samples were then reduced with 5 mM sodium borohydride for 60 min at 25°C. Biotinylated proteins were filtered through Zeba Spin Desalting Columns (Pierce, Rockford, IL) to remove excess biotin hydrazide. Biotinylated samples were then applied to a streptavidin HP Spintrap column (GE Healthcare, Piscataway, NJ) for 60 minutes at 25°C. Following 5 washes with 2.0 M urea in PBS, biotinylated proteins were eluted in 0.1 M ammonium hydroxide (5x), and samples were dried to completion using a vacuum centrifuge. Proteins were resuspended in Laemmli buffer and allowed to migrate approximately 1 cm into an SDS-PAGE gel and were Coomassie stained. The entire protein band was excised from the gel, digested with sequencing-grade trypsin (Promega, Madison, WI) in 50 mM ammonium bicarbonate overnight at 37°C following a standard digestion protocol, and the resulting peptides were analyzed by LC-MS/MS.

Peptide separation was performed by nano-Advance Splitless nano-LC at a flow rate of 500 nL/min with a gradient of 5 to 45% solvent B (90% acetonitrile, 0.1% formic acid) over 120 min on a 0.1 mm × 150 mm Magic AQ C18 column (Michrome). The LC was coupled to an amaZon speed ETD ion trap mass spectrometer with captive spray ion source (Bruker Daltonics, Inc., Billerica, MA). The instrument was operated using data-dependent collision-induced dissociation (CID) MS/MS with a threshold for fragmentation at 100,000 counts (TIC). Data analysis was performed using Mascot (v 2.4, http://www.matrixscience.com) and Proteinscape (Bruker Daltonics). Peptide identifications were accepted if they could be established at greater than 95.0% probability, and protein identifications were accepted if they could be established at greater than 99.0% probability. All samples were run in duplicate. Searches resulted in the assignment of a UniProt (Universal Protein Resource, http://www.uniprot.org/) accession number that was used for subsequent bioinformatics analysis in ExPASy (http://www.expasy.org).

### 2.7. Functional Annotation and Enrichment

The carbonylated proteins identified in two mouse brain regions and the four treatment groups (8 protein lists) were functionally annotated using the Database for Annotation, Visualization, and Integrated Discovery (DAVID, version 6.7) [37, 38] based on UniProt IDs. KEGG pathways were examined for overrepresentation in carbonylated protein lists. KEGG pathways that were nominally significant (*P* < 0.01) in at least one of the 8 protein lists and not in all 8 protein lists were used in further analyses. An enrichment *P* value was not calculated for an individual pathway if the protein list being analyzed did not contain at least two proteins from the pathway. When this occurred, a *P* value of 1 was assumed. Hierarchical clustering was used to describe relationships among enriched pathways with respect to significance in the 8 protein lists; that is, pathways enriched in the same treatment groups and brain regions were clustered together. The distance measure for hierarchical clustering was calculated as one minus the Spearman correlation coefficient when *P* values were converted to a binary indicator of significance (*P* < 0.01 versus *P* ≥ 0.01). The hierarchical clustering analysis and the heatmap were generated using R Statistical Software (version 3.1.1).

## 3. Results

### 3.1. Locomotor Activity Was Not Affected by Pesticide Treatment

**Figure 1:**
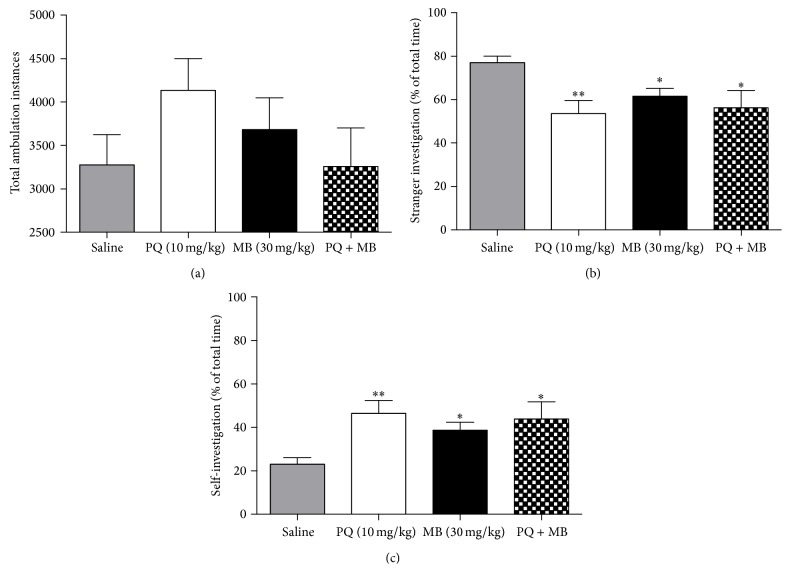
Pesticide treatment significantly alters olfactory discrimination, but not locomotor activity. PQ treated mice exhibited hyperactivity; however, these changes were not significant (a). Olfactory discrimination experiments revealed that all treatments caused a decrease in stranger investigation (b) and increased investigation of self (c). Data (mean ± SEM) analyzed via one-way ANOVA and Dunnett's post hoc test (^*∗∗*^ *P* < 0.01; ^*∗*^ *P* < 0.05). *N* = 10–12 animals per treatment group.

Previous studies employing this similar dosing regimen have reported deficits in motor function; therefore, we examined the animals for altered locomotor activity. The entire observation period was analyzed by hour, and no significant treatment-related decreases in locomotor activity were observed compared to saline controls. PQ treated animals did display hyperactivity in the first hour of testing; however, this observation was not statistically significant. In addition, the total number of ambulation instances did not differ significantly between treatment groups (Figure 1(a)). Nonmotor symptoms, such as olfactory problems, also occur in PD; therefore, we next investigated pesticide-mediated effects on olfactory discrimination.

### 3.2. Pesticide Treatment Significantly Altered Olfactory Discrimination

A deficit in olfactory function is a hallmark clinical feature of PD. Interestingly, all pesticide treatments (PQ, MB, and PQMB) significantly affected olfactory discrimination as evidenced by reduced investigation of strangers (Figure 1(b)) and increased self-investigation time (Figure 1(c)). Previous work from our group has demonstrated the importance of striatal dopamine levels in mediating normal olfactory function [32, 39]. Thus, we next investigated whether exposure to PQ and MB could affect dopamine content in the striatum.

### 3.3. The Combination of PQ and MB Significantly Decreased Striatal DA and the Respective Metabolites (DOPAC and HVA)

**Figure 2:**
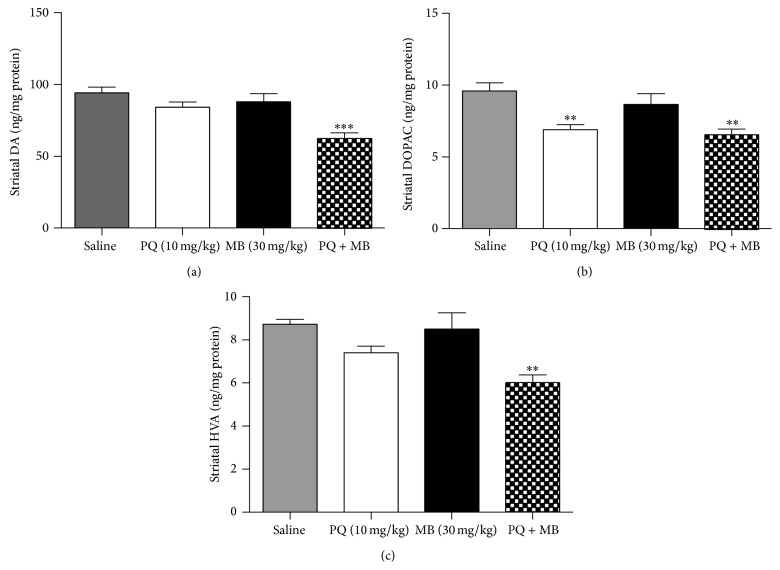
The combination of PQ and MB significantly decreases striatal DA, as well as its downstream metabolites. DA (a), DOPAC (b), and HVA (c) were quantified in striatal samples using HPLC and electrochemical detection. Data analyzed via one-way ANOVA and Dunnett's post hoc test (^*∗∗*^ *P* < 0.01; ^*∗*^ *P* < 0.05). *N* = 6–8 animals per treatment group.

Alterations in DA metabolism havebeen reported previously using this* in vivo *model [9–11, 40], and our behavioral studies show olfactory deficits; therefore, we investigated the striatal levels of DA and its proximal metabolites, DOPAC and HVA. The combination treatment (PQMB) reduced DA levels and its metabolites (DOPAC and HVA) in the striatum (Figure 2). PQ also reduced DOPAC. MB alone did not alter the striatal concentration of any of the compounds tested.

### 3.4. Extracts of Striatum and Cortex Displayed Distinct Patterns and Intensity of 4HNE Protein Adducts

**Figure 3:**
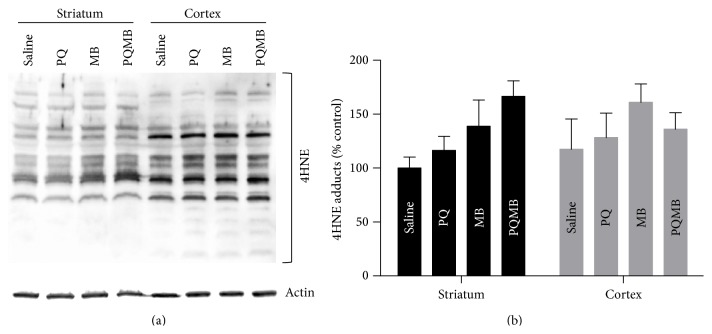
Extracts of striatum and cortex display distinct patterns and intensity of 4HNE protein adducts. Extracts (*N* = 3) were immunoblotted using an antibody specific for 4HNE-modified protein (a). Quantification of 4HNE-modified proteins shows treatment-related changes in striatal HNE adducts (b). Western blots were conducted in triplicate.

The alterations to striatal DA observed in our model could be the result of more subtle damage to the function of the dopamine terminals in the striatum that are not easily detected by immunoblot and stereology. The generation of ROS is a hallmark of PD pathology and has been shown to affect specific aspects of neuronal function [41, 42]; therefore, we aimed to assess mechanisms that may alter nerve function, such as pesticide-mediated oxidative protein adducts. To investigate this, extracts from the striatum (known to be affected by PQMB) and cortex (not known to be altered) (*n* = 3/treatment) were immunoblotted for 4HNE-modified proteins (Figure 3(a)). The staining pattern was distinct for each brain region and, compared to the striatum, the cortex displayed a much more intense staining for all the 4HNE bands observed. This translates into an increased quantity of 4HNE-adducted proteins basally present in the cortex compared to the striatum. Additionally, this blot shows that the striatum exhibits treatment-related increases in adducts compared to the cortex (Figure 3(b)). 4HNE protein adducts represent one of many possible protein carbonylation products; therefore, proteomics investigations were performed on the cortex and striatum of these mice to identify carbonylated proteins and to elucidate the metabolic pathways affected.

### 3.5. Proteomic Investigation of Carbonylated Proteins Showed Toxicant Specificities for Brain Regions

**Figure 4:**
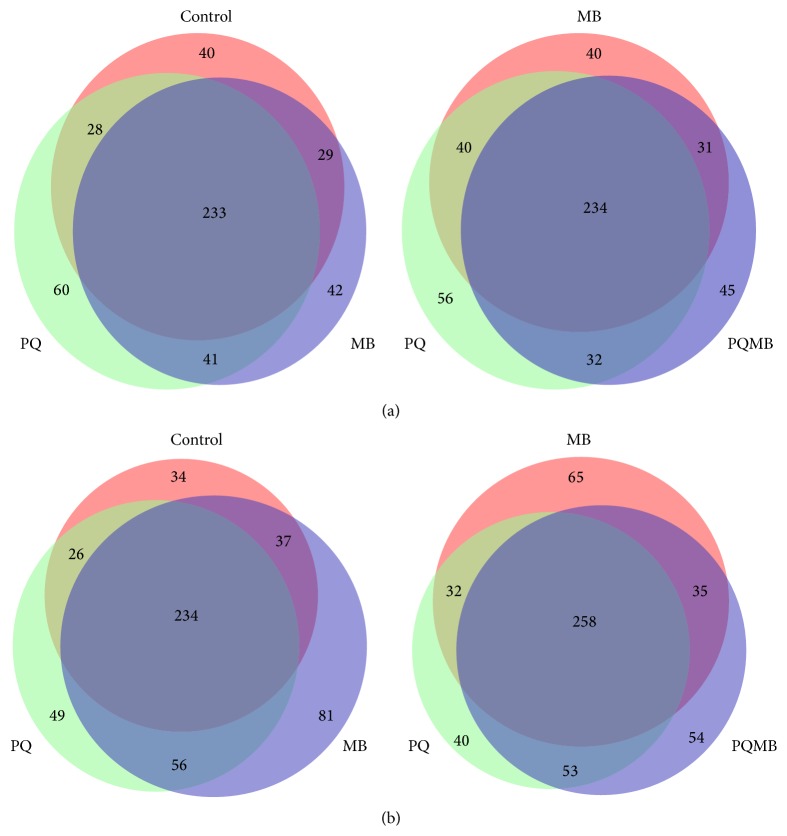
Proteins detected in each brain region display toxicant specific effects. Venn diagrams display the number of proteins that were identified to be unique or shared by the treatment groups in the striatum (a) and the cortex (b).

To enrich our samples for carbonylated proteins, a pull-down assay utilizing biotin hydrazide conjugation and streptavidin purification was utilized. Eluates from the pull-down were then digested with trypsin and analyzed via LC-MS/MS. Results of the proteomics analyses of the striatum revealed that a greater number of carbonylated proteins were detected in the PQ treated samples (364) compared to saline (332), MB (346), and PQMB (342) (Figure 4(a)). With respect to a comparison of saline versus PQ, MB, or PQMB treatment, PQ treated animals had the largest number of proteins unique to the treatment. Analyses of cortex samples were slightly different when compared to the striatum. A greater number of carbonylated proteins were detected in MB-treated samples (408) compared to saline (332), PQ (367), and PQMB (400) (Figure 4(b)). Similar to PQ in the striatum, MB-treated animals had the largest number of unique proteins (81) compared to the other conditions. These results demonstrate that PQ has greater effects on the striatum, while MB alters the cortex to a larger degree.

### 3.6. Bioinformatics Analyses Showed That Protein Carbonylation Affects Cellular Junctions, Cytoskeleton, and the Proteasome 

**Figure 5:**
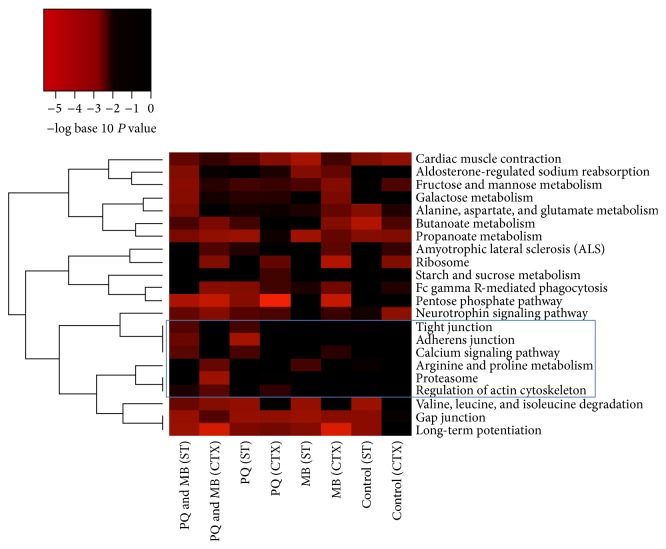
KEGG pathways enriched for carbonylated proteins by pesticide treatment and brain region. The carbonylated proteins identified in two mouse brain regions, striatum (ST) and cortex (CTX), were functionally annotated using DAVID Tools. KEGG pathways were examined for overrepresentation in carbonylated protein lists obtained for each of the four treatment groups, maneb (MB), paraquat (PQ), paraquat and maneb combined (PQMB), and controls, and in each brain region separately. KEGG pathways that were nominally significant (*P* < 0.01) in at least one of the 8 protein lists and not in all 8 protein lists are included in the graphic. The colors of the heatmap range from white (*P* > 0.01) to bright red based on the negative log base 10 transformation of the *P* value. A *P* value of 1 was used when the KEGG pathway was represented by less than two proteins in the list. KEGG pathways (rows) are ordered based on hierarchical clustering where the distance measure was calculated as one minus the Spearman correlation coefficient when *P* values were converted to a binary indicator of significance (*P* < 0.01 versus *P* ≥ 0.01).

To gain further insight into the molecular processes altered due to carbonylation, protein lists from each treatment group were analyzed using a freely available bioinformatics program, the Database for Annotation, Visualization, and Integrated Discovery (DAVID) [37, 38]. The majority of the carbonylated proteins in each of the 8 experimental conditions (average = 96.6%) were found in the DAVID database. Thirty KEGG pathways were significantly (*P* < 0.01) overrepresented in at least one of the eight protein lists. Of the 30, 8 KEGG pathways were overrepresented in all 8 protein lists and were excluded from further analyses. The other 22 KEGG pathways were clustered based on their *P* values across the 8 protein lists (Figure 5). Hierarchical clustering analysis revealed two major clusters of KEGG pathways. One cluster (top) contains 7 pathways, while the second cluster (bottom) contains 15 pathways. This top cluster of 7 pathways consists of pathways detected in almost all of the treatments and controls. The second cluster contains pathways that were not enriched in the controls. Specifically, the boxed area shows 6 pathways significantly enriched in the PQMB group in both striatum and cortex, with little or no enrichment in the saline treated mice. Pathways with poor enrichment in the saline controls included tight junctions, adherens junctions, calcium signaling, Arg and Pro metabolism, the proteasome, and regulation of the actin cytoskeleton.

## 4. Discussion

The purpose of this study was to investigate a possible role for protein carbonylation in a PQ and MB model of neurotoxicity. After 6 weeks of biweekly treatments, we observed reductions in striatal levels of DA and its metabolites, alterations in olfactory discrimination, increases in 4HNE protein adducts in the cortex and striatum, and carbonylation of proteins enriched in cellular junctions, calcium signaling, and cytoskeletal pathways.

Olfactory deficits were observed in all of the pesticide-treated groups, which is the first time this phenotype has been characterized in this model. In addition to the striatum's role in olfaction, activation of the amygdala and hippocampus is intricately involved in forming olfactory memories [43, 44]. Given that these brain regions are often targeted in neurodegenerative diseases, these olfactory deficits are novel and intriguing, especially in light of that fact that altered olfactory discrimination is observed in animals with reduced monoamine storage capacity [39]. Because the striatum is involved in olfaction, our observation of increased 4HNE protein adducts and decreased dopamine levels in the striatum points to a potential role for protein carbonylation as a modifier of olfactory pathways. While decreased striatal DA was only observed in the PQMB group, all treated animals displayed olfactory deficits, implying the involvement of additional mechanisms. Finally, it should be noted that these treatments might be effective in damaging the olfactory bulb, and this mechanistic possibility will be evaluated in future studies.

A potential mechanism for these olfactory deficits may involve the cortex. Our data demonstrate distinct patterns of 4HNE-modified proteins in this brain region. Impaired olfactory discrimination has been reported in response to cortical injury and atrophy [45, 46]. Interestingly, the loss of the sense of smell is one of the earliest signs of PD, a symptom reported years before the onset of motor symptoms [47]. Additionally, olfactory deficits are being investigated as a diagnostic approach to detect and discriminate between depression and Alzheimer's disease (AD) in the elderly [44], given that olfactory deficits are also an early symptom of AD [48]. Together, these findings corroborate with PQ and MB treatments affecting neuronal pathways and point to the importance of carbonylation in the modification of neuronal activity and function.

These studies also demonstrate treatment-mediated changes in striatal DA and DA metabolite levels. This observation appears to be a pathological hallmark of this pesticide model. For example, Thiruchelvam and colleagues reported a decrease in striatal DA of approximately 20% in the first description of this model [11]. Additional studies from the Cory-Slechta laboratory and others have described decreases in striatal DA of 20–35% [8, 10, 12]. In our study, we observed that the combination treatment (PQMB) decreased the DA level, along with the corresponding metabolites DOPAC and HVA, approximately 35%. These observations support the PQMB treatment regimen as a model system for striatal DA depletion and PD.

The striatum and cortex were shown to have distinct patterns of 4HNE adducts in response to PQ, MB, and PQMB treatment. This is a novel and important observation in light of the fact that lipid peroxidation is increased in neurodegenerative diseases like PD, AD, and Down syndrome [49]. Increased protein carbonylation due to PQMB has been observed previously [7]; however, this study investigated changes in whole brain instead of the specific regions described here, and these investigators did not employ proteomics to evaluate possible proteins and metabolic pathways affected. In further support of our findings, increased 4HNE protein adducts have been detected in a* C. elegans* model of a common genetic form of PD involving the gene LRRK2 [50]. This study also supports the involvement of ROS and aldehyde adducts in the development of PD. As redefined by Jones [18], oxidative stress involves a disruption of redox signaling and control. Cellular redox signaling and control employs reactive Cys residues in proteins to act as “sulfur switches” that control a host of processes like macromolecular interactions, allosteric control, and active site “on-off” switches [51]. Previous studies have demonstrated the importance of Cys modification by 4HNE in regard to altering protein function [25–27, 52, 53]. Additionally, proper function of DAT is reliant on redox sensitive Cys residues, as evidenced by studies involving oxidants that result in Cys oxidation and conformational changes [54, 55]. VMAT2 function is also affected by modification of redox sensitive Cys residues [56]. TH activity is also inhibited by redox thiols. For example, TH activity was found to be inhibited by toxicant- or oxidative stress-mediated glutathionylation of six of seven Cys residues [57], which may account for decreases in striatal DA observed in our study. These data indicate that oxidative stress induced by pesticide exposure leads to enhanced lipid peroxidation and aldehyde production. Thus, these aldehydes and/or ROS are capable of altering redox control of enzymes and transporters responsible for proper functioning of dopaminergic neurons.

Further investigation of the proteomic data for significant KEGG pathway enrichment revealed two distinct clusters of pathways. The smallest cluster contained 7 KEGG pathways and the majority of the pathways were significantly enriched in the saline controls as well as the toxicant treated animals. This data indicates that these particular pathways are possible targets of reactive aldehydes produced during normal metabolic processes. The second cluster of pathways is more variable and contains a block of 6 pathways that have little to no significant enrichment in the saline treated controls. These pathways include tight junctions, adherens junctions, calcium signaling, Arg and Pro metabolism, the proteasome, and regulation of the cytoskeleton. Three of these six pathways, tight junctions, adherens junctions, and regulation of actin cytoskeleton, involve cytoskeletal proteins (actin) and various regulatory proteins. The actin cytoskeleton is involved in many neuronal processes such as axon growth, arborization, and synaptic function [58]. Posttranslational modifications of the cytoskeleton, such as phosphorylation and glycation, have been shown to cause alteration to axonal structure and function. These changes may lead to neuronal degeneration, inappropriate targeting of neurons, or migration issues [59, 60]. Additionally, actin is involved in maintaining the proper structure and function of dopaminergic neurons [61]. In our study, we detected carbonylation of actin isoforms (*β*-actin and *γ*-actin), as well as members of the Arp2/3 complex (ARC1A, ARPC2, and ARPC4) and RHOA and WASF1, which are involved in F-actin nucleation and axon arborization. The impaired olfactory discrimination that we observed may be a consequence of alterations in the actin cytoskeleton; however, future studies are being conducted to specifically evaluate the toxicant-mediated effects on the actin cytoskeleton and axonal/synapse morphology.

Actin and its regulatory proteins are also involved in cellular junctions, adherens and tight. Tight junctions function as “gates” that prevent paracellular passage of ions and solutes between cells [62]. The main structures responsible for the barrier properties of the blood-brain barrier (BBB) are tight junctions [63]; therefore, any unfavorable alteration in proteins involved in this function may render this barrier permeable to solutes that do not normally passively diffuse across. PQ remains a controversial toxicant in regard to its ability to traverse the BBB [64, 65]. Altered BBB function could provide an explanation for the enhanced neurotoxicity observed by previous investigators when mice are treated with PQ and MB in combination [10–12].

## 5. Conclusion

This study further demonstrates that PQ and MB treatments negatively affect the brain and central nervous system. This was evidenced by treatment-related deficits in olfactory discrimination and decreased striatal dopamine. Additionally, we showed that there are distinct patterns of 4HNE modification in the cortex compared to the striatum. Further analyses of these modified proteins revealed treatment-related enrichment in various KEGG pathways. In particular, a cluster of 6 pathways (most of which involved the actin cytoskeleton) was enriched in treated samples, but not saline controls. These data indicate possible dysfunction in actin cytoskeletal dynamics, which would lead to altered axonal growth, arborization, and synaptic function. Future experiments are required to confirm the mechanistic involvement of actin cytoskeletal dynamics in the observed pesticide-mediated neurotoxicity.
